# T-tube drainage versus choledochojejunostomy in hepatolithiasis patients with sphincter of Oddi laxity: study protocol for a randomized controlled trial

**DOI:** 10.1186/s13063-020-04483-z

**Published:** 2020-06-29

**Authors:** Jiang-ming Chen, Xi-yang Yan, Tao Zhu, Zi-xiang Chen, Yi-jun Zhao, Kun Xie, Fu-bao Liu, Xiao-ping Geng

**Affiliations:** 1 Department of Surgery, The First Affiliated Hospital of Anhui Medical University, Wanshui Road 120#, Gaoxin District, Hefei, 230022 Anhui China; 2 Department of Surgery, The Second Affiliated Hospital of Anhui Medical University, Furong Road 678#, Shushan District, Hefei, 230022 Anhui China; 3 Department of Anesthesiology, The First Affiliated Hospital of USTC, Tianehu Road 1#, Administrative District, Hefei, 230022 Anhui China

**Keywords:** Hepatolithiasis, Sphincter of Oddi, Choledochojejunostomy, T-tube drainage

## Abstract

**Background:**

Residual and recurrent stones remain one of the most important challenges of hepatolithiasis and are reported in 20 to 50% of patients treated for this condition. To date, the two most common surgical procedures performed for hepatolithiasis are choledochojejunostomy and T-tube drainage for biliary drainage. The goal of the present study was to evaluate the therapeutic safety and perioperative and long-term outcomes of choledochojejunostomy versus T-tube drainage for hepatolithiasis patients with sphincter of Oddi laxity (SOL).

**Methods/design:**

In total, 210 patients who met the following eligibility criteria were included and were randomized to the choledochojejunostomy arm or T-tube drainage arm in a 1:1 ratio: (1) diagnosed with hepatolithiasis with SOL during surgery; (2) underwent foci removal, stone extraction and stricture correction during the operation; (3) provided written informed consent; (4) was willing to complete a 3-year follow-up; and (5) aged between 18 and 70 years. The primary efficacy endpoint of the trial will be the incidence of biliary complications (stone recurrence, biliary stricture, cholangitis) during the 3 years after surgery. The secondary outcomes will be the surgical, perioperative and long-term follow-up outcomes.

**Discussion:**

This is a prospective, single-centre and randomized controlled two-group parallel trial designed to demonstrate which drainage method (Roux-en-Y hepaticojejunostomy or T-tube drainage) can better reduce biliary complications (stone recurrence, biliary stricture, cholangitis) in hepatolithiasis patients with SOL.

**Trial registration:**

Clinical Trials.gov: NCT04218669. Registered on 6 January 2020.

## Background

Hepatolithiasis (HL), defined as the occurrence of stones in any intrahepatic bile duct proximal to the confluence of the right and left hepatic ducts, is a common disease in Southeast Asia and accounts for up to 20% of all cases of gallstone disease. Although rare in Western countries, with a reported incidence of 0.6 to 1.3%, an increasing trend has been observed due to immigration from endemic areas and the westernization of diets [1]. HL is benign in nature, but without appropriate treatment, it can lead to repeated cholangitis, liver abscesses, biliary strictures, secondary biliary cirrhosis and even cholangiocarcinoma [2].

The principles of HL management are clearance of stones, correction of strictures, removal of non-functioning liver segments that harbour bacteria and serve as foci of infection, and restoration of bile drainage [3]. The curative management of HL is difficult since the pathogenesis of this condition is not completely understood, and the best definitive management of HL is a multidisciplinary approach that consists of laparoscopic, open, endoscopic and percutaneous surgical approaches. However, residual and recurrent stones remain one of the most important challenges of HL and are reported in 20 to 50% of patients treated with these therapies. The risk factors associated with stone recurrence were reported to be liver atrophy, bile duct stricture, dilatation, residual stones and sphincter of Oddi laxity (SOL) [4, 5].

SOL results in the reflux of duodenal fluid and enteric bacterial infections, which lead to the formation of stones in the biliary tract. Roux-en-Y hepaticojejunostomy (HJ) shows considerable advantage in preventing the reflux of intestinal contents into the bile duct [6, 7]. There is no consensus on which drainage method is better for HL patients with SOL, and there are no prospective randomized controlled studies that have evaluated the effects of different drainage methods. As a result, we decided to carry out a randomized controlled trial (RCT) to evaluate the therapeutic safety and perioperative and long-term outcomes of HJ versus T-tube drainage for HL with SOL.

## Methods/design

### Study design

**Fig 1:**
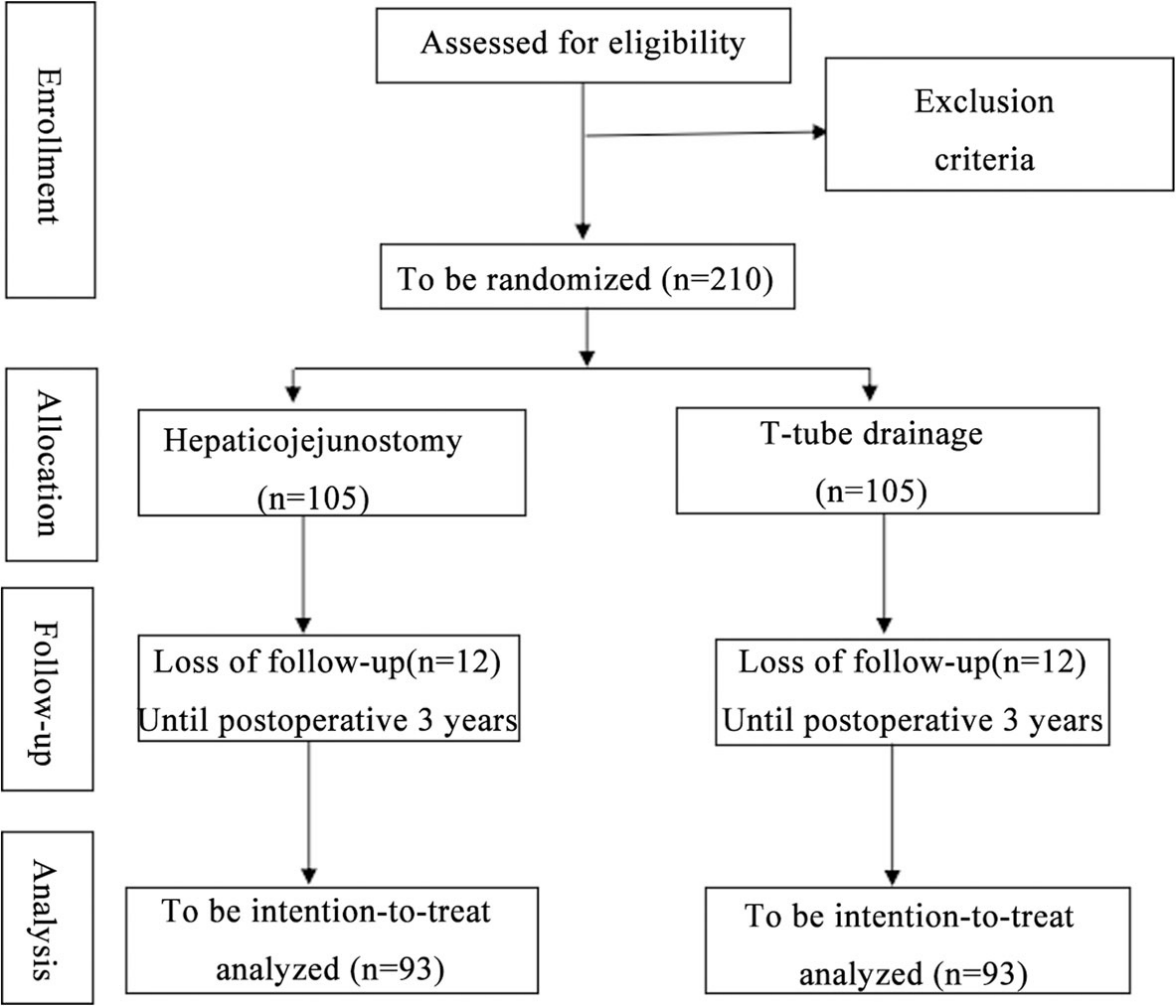
Flowchart according to CONSORT

**Fig 2:**
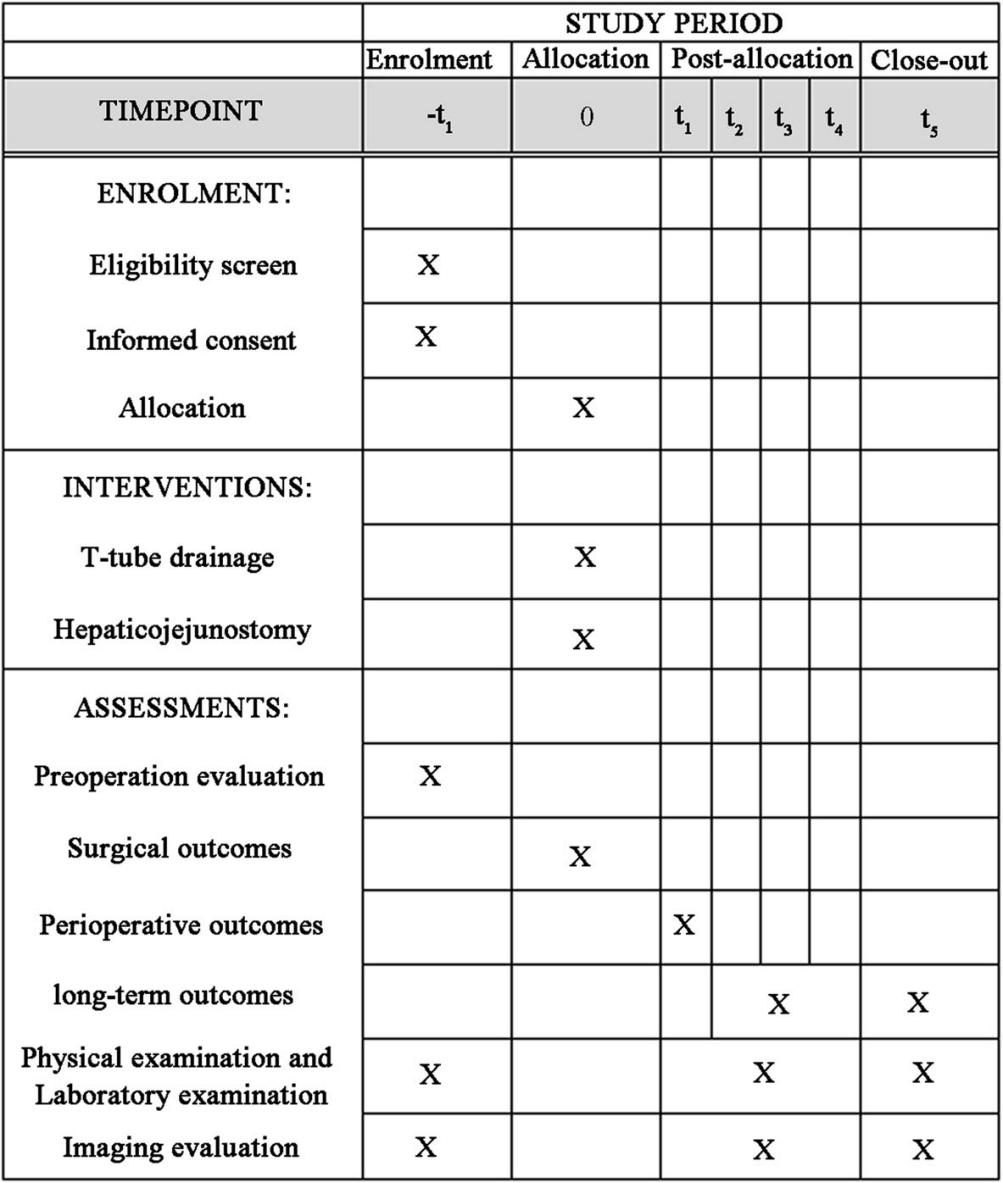
Schedule of enrollments, interventions and assessments. Abbreviations: t_1_, 3 months after surgery; t_2_, 6 months after surgery; t_3_, 1 year after surgery; t_4_, 2 years after surgery; t_5_, 3 years after surgery

This prospective, single-centre, randomized and interventional clinical trial is being conducted at the First Affiliated Hospital of Anhui Medical University, which began in February 2020 and is expected to end in 2026. Patients with diagnosed HL who meet the entry criteria will be recruited and randomly assigned to undergo either T-tube drainage or Roux-en-Y HJ. The flowchart according to the Consolidated Standards of Reporting Trials (CONSORT) statement is shown in Fig. 1, and the schedule of enrolment, interventions and assessments are summarized in Fig. 2. The Standard Protocol Items: Recommendations for Interventional Trials (SPIRIT) guidelines are provided in Additional file 1. The full protocol and the datasets analysed during the current study are available on request from the corresponding author.

### Number of patients needed

The sample size is based on a two-sided *t* test for differences with respect to the primary parameter and the primary analysis. In a retrospective analysis of our own series, the incidence of biliary complications was 30% in patients with HL with SOL undergoing T-tube drainage. The calculated sample size was 186 (93 per group), with 80% statistical power to detect a difference in the supposed incidence of biliary complications of 15% (30% in the T-tube drainage group versus 15% in the Roux-en-Y HJ group). Given an estimated withdrawal rate of 10%, the minimum required sample size would be 204 patients. We plan to enrol a total of 210 patients (105 per group).

### Eligibility criteria

#### Inclusion criteria

The following are the inclusion criteria:

1. Aged between 18 and 70 years
2. Diagnosed with HL with SOL during the operation
3. Underwent foci removal, stone extraction without residue and stricture correction during the operation
4. Provided written informed consent
5. Was willing to complete a 3-year follow-up.

#### Exclusion criteria

The following are the exclusion criteria:

1. Participation in concurrent intervention trials that would interfere with the outcomes of this study
2. Associated tumour
3. Complete loss of function or normal sphincter of Oddi (SO)
4. Previous choledochojejunostomy
5. Intraoperative stone residual
6. Lack of compliance

### Withdrawal

An informed consent form, which describes the detailed study procedures and illustrates the potential benefits and risks, will be provided to all participants so that they can decide whether to volunteer. All the participants are freely informed to participate in the study and can withdraw from the trial at their own request at any time. If the patient withdraws, the information will not be used in this study. However, the research team can still record the outcome data in clinical report forms.

### Ethics, study registration and consent

The final protocol was approved by the Ethics Committee of the First Affiliated Hospital of Anhui Medical University (PJ2019-17-12). The trial protocol was registered at the ClinicalTrials.gov protocol registration system (NCT04218669). Patients with HL who are scheduled for common bile duct exploration at the Department of General Surgery, Anhui Medical University, will be screened for eligibility and informed about the trial. The study procedure, benefits, risks and data management will be clarified in detail during the conversation.

### Trial interventions

All patients with SOL underwent common bile duct exploration and complete stone extraction. The surgical procedures of the T-tube drainage arm and Roux-en-Y HJ arm are as follows.

#### Group A: T-tube drainage

A T-tube was placed for biliary drainage, and the common bile duct was intermittently sutured with 4-0 Vicryl sutures.

#### Group B: Roux-en-Y HJ

The common hepatic duct was cut, and the duodenal side was closed with sutures. The small intestine was cut off 15 cm below the ligament of Treitz. The distal end was lifted, and a 1–2-cm incision was made at the jejunal wall 4–5 cm from the jejunal stump. The anastomosis used 5-0 PDS II with double-armed inside-out sutures in the jejunum and outside-in sutures in the hepatic duct. One side of the needle was used to continuously penetrate and suture the whole layer of the posterior lateral wall of the jejunum and the posterior lateral wall of the biliary duct, and the other side of the needle was used to continuously suture the anterior part of the anastomosis. Mucosa-to-mucosa contact should be ensured with every stitch. The anastomotic stomas were then checked for leakage. The enteric-enteric anastomosis was performed 60 cm below the site of the hepaticojejunal anastomosis.

### Primary and secondary endpoints

All participants will be followed up regularly according to the schedule shown in Fig. 2 at the stated intervals after surgery for at least 3 years. At the follow-up visits, patients will undergo laboratory tests and abdominal ultrasonography as described in Fig. 2. CT or MRI will be performed in cases of suspected stone recurrence or cholangitis. Laboratory tests mainly include routine blood, liver and kidney function, electrolytes, procalcitonin, C-reaction protein, carcinoembryonic antigen, carbohydrate antigen 199 (CA199) and alpha-fetoprotein (AFP).

The primary efficacy endpoint of the trial will be the incidence of biliary complications (stone recurrence, biliary stricture, cholangitis). Stone recurrence will include any bile duct stones detected with any imaging modality during follow-up. Biliary stricture will be defined as clinically evident stenosis and subclinical stenosis proven by endoscopic examination or reoperation. The diagnosis of cholangitis will be based on clinical evidence (abdominal discomfort/pain, jaundice or fever associated with hepatolithiasis).

The secondary outcomes will be the surgical, perioperative and long-term follow-up outcomes. The surgical outcomes will include operative duration, intraoperative blood loss, intraoperative blood transfusions, stone distribution and SO function. The perioperative events occurring within 90 days after surgery will be recorded, including biliary leakage, wound infection, pulmonary infection, reoperation, hepatic injury, haemorrhage, bowel function recovery, intra-abdominal fluid collection or abscess, mortality, duration of postoperative hospital stay and total hospitalization expenditure. The indices of long-term follow-up outcomes will be hepatic injury, stone recurrence, biliary stricture, rate of unplanned readmission for HL, incidence of cholangitis and quality of life. Postoperative complications will be graded based on severity according to the Clavien-Dindo definition (shown in Additional file 2: Table S1) [8]. Long-term quality of life will be assessed by clinical grading according to Terblanche et al. [9]. Grades I and II constituted excellent or good results, grade III fair and grade IV poor (shown in Additional file 2: Table S2).

Laboratory examination routinely collects patient blood samples in the ward or clinic. The blood samples store at room temperature and test in the laboratory of our hospital that day. The specific time laboratory examination and abdominal ultrasonography are shown in Fig. 2, and the perioperative laboratory examination time is the first day, the third day, and the seventh day after the operation.

### Randomization and blinding

To achieve comparable groups of known and unknown risk factors, randomization will be performed. Patients meeting the eligibility criteria will be randomly assigned (1:1) to the T-tube drainage arm or Roux-en-Y HJ arm by computer-generated allocation based on the envelope method and the hierarchical block randomization method. The envelopes will be opened after common bile duct exploration.

Blinding of the surgeons and patients is not feasible due to the obviously different characteristics of the two types of biliary drainage. However, the statisticians will be blinded to the treatment allocation during data collection and analysis.

### Standardization of perioperative care

**Fig 3:**
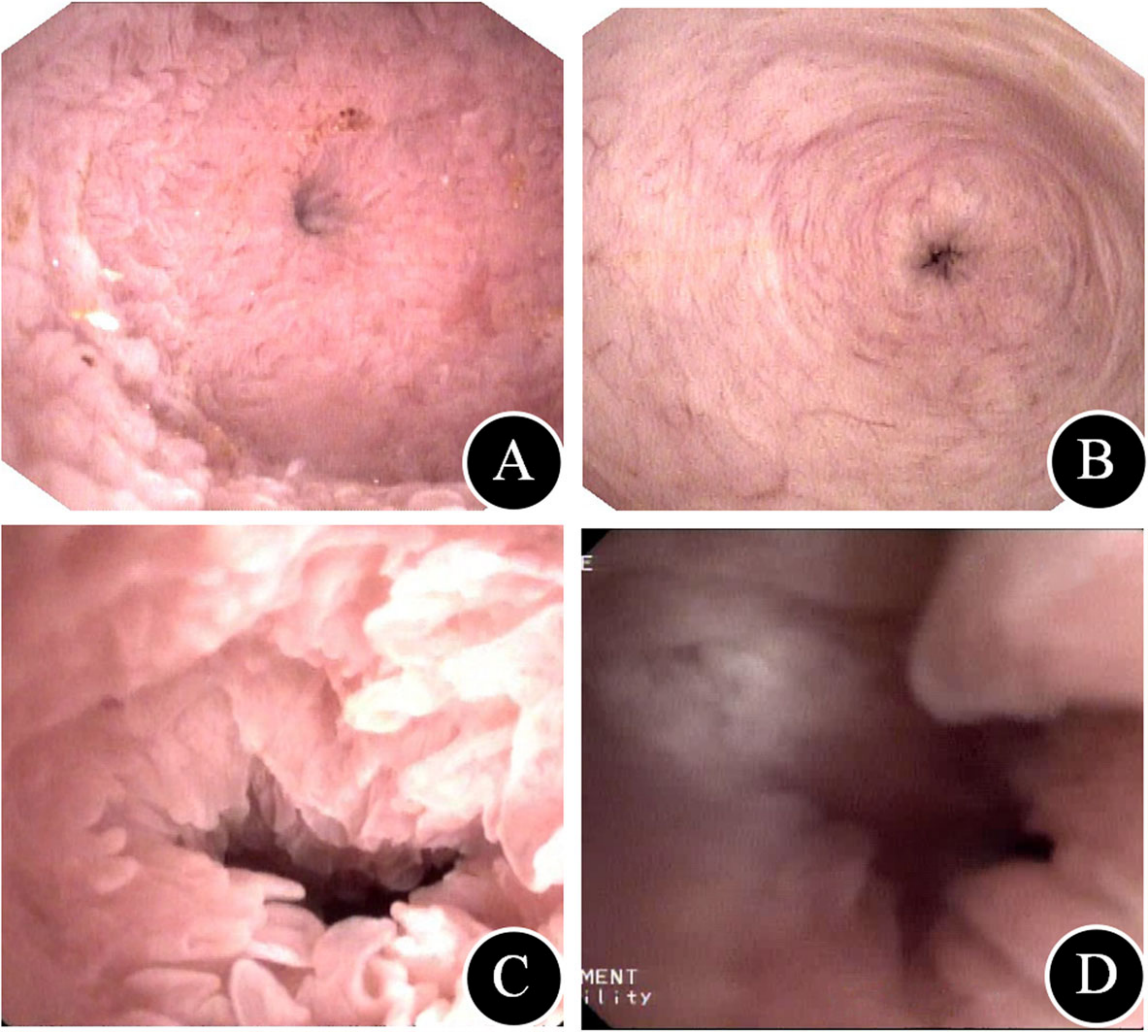
Shape of the sphincter of oddi. **a** Radial form. **b** Comma form. **c** Oval form. **d** Irregular form

The operations will be performed by two senior surgeons (XP Geng and FB Liu) who are equally skilled in T-tube drainage and Roux-en-Y HJ. Intraoperative flexible choledochoscopy (CHF-P20, external diameter, 4.9 mm; Olympus, Tokyo, Japan) will be routinely performed to explore the biliary ducts, identify the function of the SO and remove residual stones. All patients included in the study should successfully undergo cleaning of all ductal stones, removal of all strictures and elimination of the non-functioning liver segments that harbour bacteria and serve as foci of infection. For the patients with T-tubes, cholangiography will be performed to ensure that all the stones are clean before the T-tubes are removed. If there are residual stones, they could be cleaned through postoperative choledochoscopy. Indications for liver lobe resection will include a liver segment or lobe filled with stones that were inaccessible by other approaches for treatment, strictures associated with stones, atrophy of the affected liver segments or lobe, presence of liver abscess, or suspected cholangiocarcinoma. The selection criteria for hepatectomy will require patients to have Child’s A grade liver function and no evidence of portal hypertension. The grading criteria for SO function are as follows: normal—the shape of the SO is circular (Fig. 3a) or comma-like (Fig. 3b) with normal rhythmic contractions, a flexible choledochoscope cannot enter the duodenum and the SO can completely close without gaps during closure; SOL (shown in Additional file 3: Video S1-Grading criteria for the SO function laxity)—the shape of the SO is oval (Fig. 3c) or irregular (Fig. 3d), a flexible choledochoscope can enter the duodenum with some resistance, the SO cannot completely close during closure or reflux of food or methylene blue can be seen in the common bile duct during flexible choledochoscopy; and loss of function—the flexible choledochoscope can directly enter the duodenum, and no contractions are observed after the morphine-neostigmine test.

**Additional file 3.** Grading criteria for the SO function laxity.

### Provisions for post-trial care

There is no anticipated harm and compensation for trial participation

### Data management and quality assurance

The principal investigators and data analysts will have access to the final cleaned dataset. All paper forms will be scanned and stored at the password cabinet in the First Affiliated Hospital of Anhui Medical University office of clinical trials. All electronic data will be coded and stored separately without the personal data in on a password-protected computer. The files of the participants will be stored for a minimum of 10 years after the end of the study. All relevant information for each subject should be recorded in the case report form (CRF) and inputted into the Microsoft Access database in a timely, truthful and precise manner by trained research staff. An explanation should be given for all missing data. Complete CRF pages will be checked by the principal investigator and the responsible monitor with respect to completeness and plausibility. The patients will also be informed of the outpatient follow-up interviews by telephone, and all items will be regularly accordingly assessed to the schedule shown in Fig. 2. Changes to the database can only be made with joint written consent from the data managers, statisticians and clinical research leaders. The results will be submitted for publication in peer-reviewed journals.

To ensure the safety and validity of the trial, the data will be overseen by an independent Data Safety Monitoring Board (DSMB) for efficacy outcomes and safety, which consists of surgical experts, statistics experts and ethics experts. It is independent from the sponsor and competing interests. During the trial, the DSMB is charged with providing advice recommendations that include (a) continuation of the study, (b) continuation with modification and (c) termination of the study.

### Confidentiality

All data will be treated as confidential. The data will be acquired only by researchers, research institutions and ethics committees who have signed a confidential disclosure agreement. The study will not release the individual identities of the participants without consent, except in special circumstances as required by law.

### Statistical analysis

The two-sided null hypothesis for the primary outcome measure states that both study interventions lead to a similar rate of stone recurrence; the alternative hypothesis is that one intervention will perform better than the other. The continuous variables will be recorded as the mean ± SD (for normally distributed variables) or medians and interquartile ranges (for skewed parameters) and will be statistically analysed with Student’s *t* test and the non-parametric Mann-Whitney *U* test, respectively.

Categorical data will be presented as frequencies and group percentages and compared with Fisher’s exact test. Graded data, including age, quality of life and Clavien-Dindo classification, will be compared with the Wilcoxon signed-rank test.

The odds ratio for having biliary complications will be determined with a logistic regression model. The homogeneity of the two groups will be described by comparing the demographic data and the baseline values.

All analyses will be performed on an intention-to-treat (ITT) basis. Statistical Package for the Social Sciences (SPSS) 10.0 (SPSS Inc., Chicago, IL, USA) will be used for statistical analysis. Statistical significance will be defined as *P* < 0.05.

## Discussion

HL is characterized by its intractable nature and frequent recurrence. The risk factors associated with stone recurrence were reported to be liver atrophy, bile duct stricture, dilatation, residual stones and SOL [2]. This study aimed to demonstrate which drainage method (Roux-en-Y HJ or T-tube drainage) is better for reducing biliary complications in HL patients with SOL.

In 1887, Ruggero Oddi, an Italian anatomist, first proposed the concept of the duodenal papillary sphincter, which was later confirmed and named the SO. The SO is a muscular structure surrounding the confluence of the distal common bile duct and the pancreatic duct into the ampulla of Vater. The main function of the SO is to regulate bile flow into the duodenum and prevent duodenal reflux. With SOL, the SO will undoubtedly lose its one-way “gate” function, which can cause severe bile-intestinal reflux and biliary tract infection [10]. For patients with normal SO function, the best biliary drainage is T-tube drainage. T-tube drainage is relatively simple and has a high stone clearance rate. Because this method retains SO function, it also retains the integrity and continuity of the extrahepatic bile duct structure. T-tube drainage can significantly reduce the incidence of postoperative reflux cholangitis in patients with normal SO function. For patients with complete loss function or SO stenosis, Roux-en-Y HJ is currently one of the best biliary drainage methods [10]. The advantage of Roux-en-Y HJ is that it reduces the reflux of duodenal fluid, but this operation abandoned the Oddi sphincter. The results of a retrospective study showed that Roux-en-Y HJ can significantly reduce the incidence of postoperative cholangitis and recurrence of stones in patients with SO dysfunction [11–13].

There is no consensus on which drainage method is better for HL patients with SOL, and there are no prospective randomized controlled studies that have evaluated the effects of different drainage methods. The biliary drainage method is chosen arbitrarily and is mostly based on the surgeon’s habits. When performing bile duct exploration during surgery, most surgeons often judge the function of the sphincter with a manometry probe or catheter and determine which biliary drainage method is needed. These methods are mostly empirical, lack scientific evaluation and cannot accurately reflect the true function of the SO. This study design features methods and evaluation criteria to evaluate the SO function during the operation. In this prospective randomized controlled study, the patients will be divided into the HJ group and the T-tube drainage group. The postoperative follow-up is standardized. Through statistical analysis of the results of the two groups, a systematic evaluation can be performed to recommend the best surgical method for HL patients with SOL. We believe that the results of this trial will significantly contribute to the evidence on which biliary drainage method is better for HL patients with SOL.

## Trial status

The study protocol was completed in December 2019. Enrolment started on 1 February 2020. The study will continue until sufficient power is reached, approximately until December 2026. Protocol version n.3, January 2020.

## Supplementary information

**Additional file 1.** SPIRIT 2013 checklist: recommended items to address in a clinical trial protocol and related documents.

**Additional file 2: Table S1.** The Clavien-Dindo Classification of postoperative complications. **Table S2.** Clinical Grading of Long-term Quality of Life.

## Data Availability

Datasets used or analyzed in the current study may be provided upon reasonable request of the corresponding author.

## Abbreviations

HL: Hepatolithiasis
SOL: Sphincter of Oddi laxity
HJ: Hepaticojejunostomy
SO: Sphincter of Oddi
CRF: Case report form
