# Supplementary material for: T-tube drainage versus choledochojejunostomy in hepatolithiasis patients with sphincter of Oddi laxity: study protocol for a randomized controlled trial
Source: Trials. 2020 Jun 29;21:586. doi: 10.1186/s13063-020-04483-z (PMC7322885; doi:10.1186/s13063-020-04483-z)
Supplement: Supplementary file 3 — Additional file 2: Table S1. The Clavien-Dindo Classification of postoperative complications. Table S2. Clinical Grading of Long-term Quality of Life. [file 13063_2020_4483_MOESM2_ESM.doc]

Table S1 The Clavien-Dindo Classification of postoperative complications

| Grade | Definition |
| --- | --- |
| Grade I | Any deviation from the normal postoperative course without the need for pharmacological treatment or surgical, endoscopic, and radiological interventions Allowed therapeutic regimens are: drugs as antiemetics, antipyretics, analgetics, diuretics, electrolytes, and physiotherapy. This grade also includes wound infections opened at the bedside |
| Grade II | Requiring pharmacological treatment with drugs other than such allowed for grade I complications Blood transfusions and total parenteral nutrition are also included |
| Grade III | Requiring surgical, endoscopic or radiological intervention |
| Grade IIIa | Intervention not under general anesthesia |
| Grade IIIb | Intervention under general anesthesia |
| Grade IV | Life-threatening complication (including CNS complications)* requiring IC/ICU management |
| Grade IVa | Single organ dysfunction (including dialysis) |
| Grade IVb | Multiorgan dysfunction |
| Grade V | Death of a patient |

CNS, central nervous system; IC, intermediate care; ICU, intensive care unit.

Table S2 Clinical Grading of Long-term Quality of Life

| Grade | Characteristics |
| --- | --- |
| I | No biliary symptoms |
| II | Transitory symptoms, currently no symptoms |
| III | Clearly related symptoms requiring medical therapy |
| IV | Recurrent stricture requiring correction or related death. |
